# Tubularized incised plate urethroplasty and grafted tubularized incised plate urethroplasty: systematic review, meta-analysis and trial sequential analysis

**DOI:** 10.1136/wjps-2023-000707

**Published:** 2024-02-21

**Authors:** Nitinkumar Borkar, Charu Tiwari, Abhijit Nair, Debajyoti Mohanty, C K Sinha, Jai Kumar Mahajan

**Affiliations:** 1 Paediatric Surgery, All India Institute of Medical Sciences-Raipur, Raipur, Chhattisgardh, India; 2 Department of Anaesthesiology, Ibra Hospital, Ibra, Oman; 3 General Surgery, All India Institute of Medical Sciences-Raipur, Raipur, Chhattisgardh, India; 4 Paediatric Surgery, St George's University Hospitals NHS Foundation Trust, London, UK; 5 Pediatric Surgery, PGIMER, Chandigarh, India

**Keywords:** Child Health, Qualitative research, Pediatrics, Evidence-Based Medicine, Congenital Abnormalities

## Abstract

**Background:**

Hypospadias is one of the most common genital birth defects. There are around 300 various techniques available for the repair of hypospadias. This study aims to compare the reported outcomes of Tubularized incised plate urethroplasty (TIP) and Grafted TIP (GTIP) repair in children undergoing primary hypospadias repair.

**Methods:**

This meta-analysisadhered to Preferred Reporting Items for Systematic Reviews and Meta-Analyses guidelines, and we framed our research question using the population, intervention, control and outcomes format. We conducted comprehensive electronic searches across various databases, employing a Boolean search strategy with predefined search terms. Only randomized controlled trials (RCTs) were included for quantitative analysis.

**Results:**

Totally, 10 RCTs met our inclusion criteria for quantitative analysis. The results indicated that urethrocutaneous fistula, glans dehiscence, and stricture rates were comparable between the two groups. The incidence of meatal stenosis was found to be significantly lower in the GTIP group with a relative risk (RR) of 0.32 (95% confidence interval (CI) 0.15 to 0.67).

**Conclusion:**

The coucomes UCF, glans dehiscence, and stricture rates were comparable between the two groups. Notably, the incidence of meatal stenosis was found to be significantly lower in the grafted TIP group. In terms of operative time, our quantitative synthesis demonstrated that the TIP group had a shorter operative time than the GTIP group with significant heterogeneity.

**WHAT IS ALREADY KNOWN ON THIS TOPIC**

- Tubularized Incised Plate Urethroplasty (TIP) and Grafted TIP (GTIP) are widely used techniques of for hypospadias repair and proven their results over the years .

**WHAT THIS STUDY ADDS**

- This systematic review and meta-analysis provide comprehensive evidence comparing TIP and GTIP in primary hypospadias repair.
- It adds quantitative insights, revealing lower meatal stenosis incidence in GTIP, shorter operative time in TIP, and comparable outcomes in other parameters.

**HOW THIS STUDY MIGHT AFFECT RESEARCH, PRACTICE, OR POLICY**

- The finding of potential benefits of GTIP in reducing meatal stenosis, may influence surgical decision-making.
- The study also emphasizes the need for further research to establish conclusive evidence, highlighting the importance of surgeon skill and the subjectivity of outcome assessment in hypospadias repair.

## Introduction

Hypospadias is defined by an ectopic opening of the urethral meatus on the ventral aspect of the penis rather than the tip. Embryologically, this occurs because of the arrest in the normal development of the penis.^1^ The incidence of hypospadias in Europe is around 18.6 per thousand, with the highest prevalence in North America and the lowest in Asia.^2^ Hypospadias correction is usually recommended between the ages of 6 and 18 months. There are around 300 methods for the surgical correction of hypospadias which evolved over a thousand years from after Christ to the modern era. Galen from the second century was the first to use the term ‘hypospadias’.^3^ Sophisticated urethral surgery was only possible after the introduction of anesthesia by Morton in 1946.^3^ In 1880, Duplay first described the tubularization of local skin over a tube,^3^ and over the next century, various techniques were introduced using local flaps and free grafts for reconstruction of the neourethra.

In 1994, Warren Snodgrass published his tubularized incised plate (TIP) urethroplasty technique.^4^ He used this technique to correct distal hypospadias with minimal chordee. This TIP technique involves a midline incision over the urethral plate from the anomalous meatus to the glans tip, which allows mobilization of the plate for tubularization. It creates a functional neourethra with a vertically oriented slit-like meatus. Soon thereafter, the Snodgrass technique became very popular as an alternative to meatal-based and onlay island flaps for distal hypospadias. However, over the years, many surgeons have noticed that the results of this technique may be compromised, especially in patients with a narrow or shallow urethral plate. TIP repair has also been reported to have complications such as meatal stenosis (MS) and urethrocutaneous fistula (UCF). In 2000, Kolon and Gonzales^5^ reported a technique using a free graft of inner prepuce to bridge the gap created by the Snodgrass incision. This showed promising results with none of the patients in their series of 32 patients developing MS or UCF. Hayes and Malone^6^ used free buccal mucosa graft instead of inner preputial skin in their patients with satisfying results; however, preputial skin is usually preferred and widely used for hypospadias repair. Later, a few authors reported encouraging results using the technique described by Kolon and Gonzales.^5^ Mouravas and Sfoungaris^7^ were the first to publish a randomized trial comparing the results of TIP with grafted TIP (GTIP) urethroplasty. They concluded that the GTIP technique had a considerably lower rate of complications than TIP. The results of TIP and GTIP repair for primary distal hypospadias correction were comparable in a prospective randomized study by Helmy *et al*.^8^ They preferred the Snodgrass technique as the procedure of choice for primary distal hypospadias correction. A prospective randomized study by Eldeeb *et al* 9 also reported equivalent results for both TIP and GTIP groups with a shorter operating time in the TIP group. In their prospective comparative study, Ahmed *et al* 10 concluded that despite being a statistically insignificant result, GTIP repair showed better clinical outcomes. The HOSE (Hypospadias Objective Scoring Evaluation) score as a measure of cosmetic outcomes was also comparable in both groups in their study.

Further objective evaluation of urethral function by urinary flow measurement after hypospadias correction by these techniques was performed by Helmy *et al* and González and Ludwikowski.^8 11^ Both techniques have proven their results in hypospadias repair. Published literature shows the relatively recent GTIP technique to be equivalent or superior to TIP in terms of operative complications, cosmesis, and functional outcomes. This quantitative analysis aimed to systematically compare the reported outcomes of TIP and GTIP in children undergoing primary hypospadias repair.

## Methods

This meta-analysis was conducted as per Preferred Reporting Items for Systematic Reviews and Meta-Analyses (PRISMA) guidelines.^12^ In the included studies, patients included children under the age of 18 years undergoing primary hypospadias repair through dorsal inlay GTIP compared with classic TIP. Primary outcomes that were assessed included UCF, MS, glans dehiscence (GD), operative time, wound infection, and success rate. Secondary outcomes included uroflow, cosmetic scores, and urethral diverticulum. We only included randomized controlled trials (RCTs) for our meta-analysis.

### Search strategy

We searched the literature with a Boolean search using the terms: Snodgrass OR TIP OR Classic Snodgrass OR TIPU AND GTIP OR DIGU OR Snodgraft OR Grafted Snodgrass OR GTAS. Databases including Scopus, Medline, CENTRAL and Google Scholar were used to identify published RCTs. For unpublished literature, we searched gray literature in the OpenGrey Database (www.opengrey.eu). Clinical trials in ClinicalTrials.gov and WHO International Clinical Trials Registry Platform (https://trialsearch.who.int/) were also included. A snowball search was also performed to identify additional literature.

### Data collection

Two review authors (NB and CT) independently screened the study titles and abstracts for inclusion as per the search strategy. Studies were categorized as either eligible, potentially eligible or not eligible. Full texts of the eligible and potentially eligible studies were all obtained. After full-text retrieval, two review authors (DM and NB) independently screened the full text and identified studies for inclusion. They also recorded the reasons for the exclusion of ineligible studies. Disagreements were resolved through discussion, and if needed, senior authors (CKS and JKM) were consulted for the final decision. The duplication of studies was carefully removed. The selection process was meticulously documented to complete a PRISMA flow diagram.

The included studies’ characteristics and outcome data were entered in a standardized data collection form. Two review authors (NB and CT) independently extracted the following study characteristics from the included studies: author details, year of publication, study setting, study design, total duration of the study, total number of patients, number of patients in each group, demographic details, follow-up period and reported primary and secondary outcomes. A third author (AN) verified the accuracy of the data mentioned in the data chart.

### Trial sequential analysis

Trial sequential analysis (TSA) was conducted using V.0.9.5.10 of the TSA Module, developed by the Copenhagen Trial Unit in Denmark.^13^ This analysis aimed to assess the robustness of our findings. We used a fixed-effects model with the DerSimonian-Laird method to construct a cumulative Z curve. The TSA was implemented to maintain the overall risk of committing a type I error at 5%. The key determinant was whether the cumulative Z curve intersected the trial sequential monitoring boundary or entered the futility zone. Such an occurrence would indicate that there was sufficient evidence to either accept or reject the anticipated intervention effect, rendering further research unnecessary. Conversely, if the Z curve did not cross any boundaries and failed to reach the required information size (RIS), it would signify that the evidence was insufficient to draw a definitive conclusion, thus necessitating further research.

### Assessment of risk of bias

Two review authors (NB and DM) independently assessed the risk of bias for each included study using the Risk of Bias visualization (robvis) tool (RoB 2).^14^

The risk of bias was assessed according to the following domains:

- Bias arising from the randomization process.
- Bias due to deviations from intended intervention.
- Bias due to missing outcome data.
- Bias in measurement of the outcome.
- Bias in selection of the reported result.

### Measures of treatment effect

We analyzed dichotomous data risk ratios (RRs) with 95% CIs and continuous data as the mean differences (MDs) with 95% CIs.

### Assessment of heterogeneity

The observed diversity in participant characteristics and outcomes in our studies determined the clinical heterogeneity of this analysis. We used the *I* ^*2*^ statistic to measure heterogeneity among the studies in each analysis. As recommended in the Cochrane Handbook of Systematic Reviews of Interventions recommendations,^15^ an *I* ^*2*^ value of 0–40% ‘might not be important’; 30–60% may represent ‘moderate heterogeneity’; 50–90% may represent ‘substantial heterogeneity’; and 75–100% represents ‘considerable heterogeneity’ was followed in our study.

## Results

### Study characteristics

**Figure 1:**
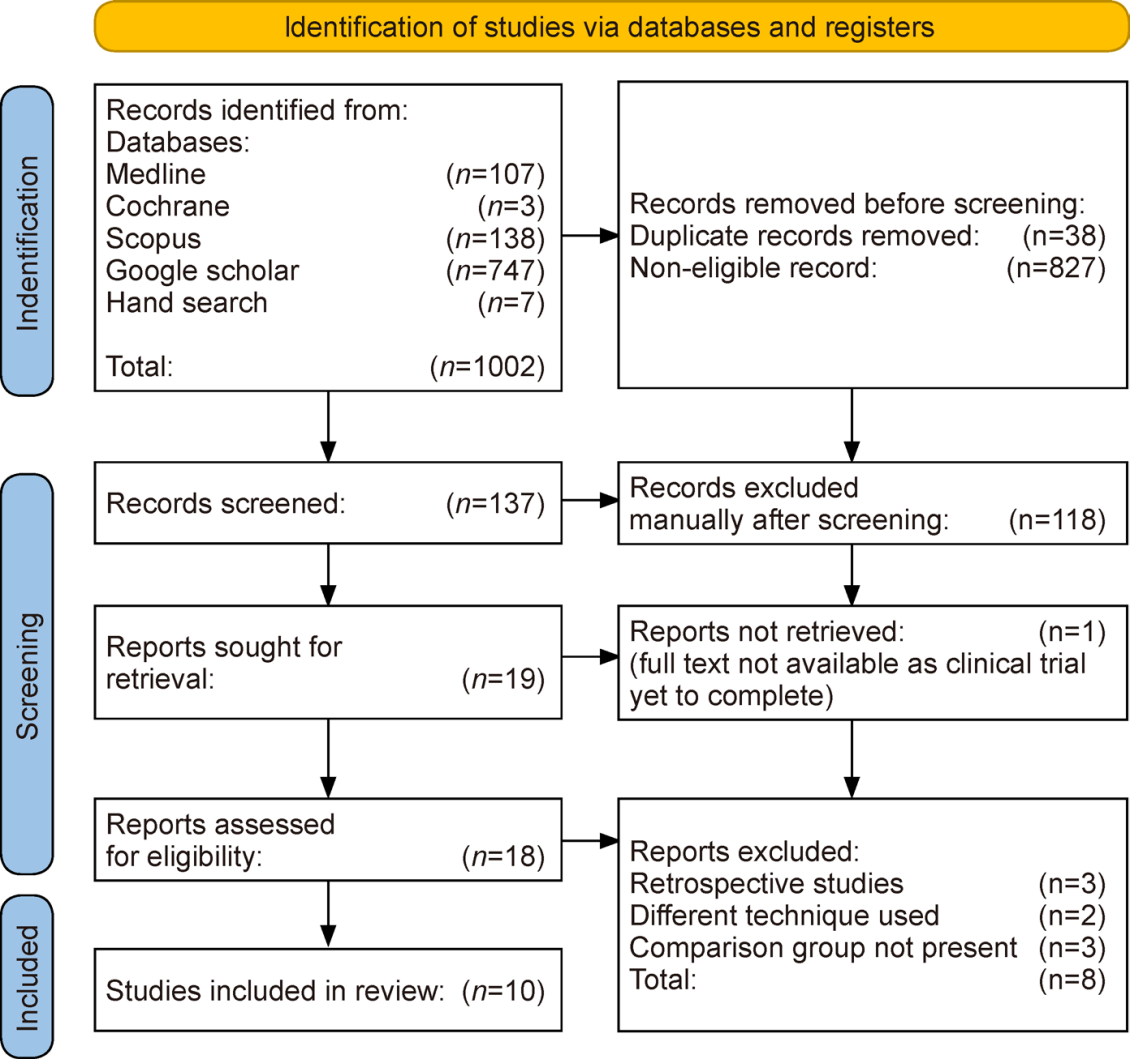
PRISMA Flow Diagram.

Our initial literature search was as per Boolean search results in 1002 articles. After removing the duplicates and excluding non-eligible studies, 10 randomized studies including 603 children^7–10 16–21^ were found to be eligible. The details of the excluded studies are also depicted in the PRISMA flow diagram (figure 1).

The characteristics of all the included studies are mentioned in table 1. Nine studies were available in English literature, and one was in Chinese with abstracts in English. In the studies, there were 300 patients in the TIP group and 303 patients in the GTIP group. The primary and secondary outcomes of all studies are mentioned in table 2.

**Table 1:** Study characteristics

	Studies	Setting	Study period	Design	Patients (n)	Mean age	Type of hypospadias	F/U (months)	Reported outcomes
1	Mouravas *et al* (2013)	Greece	March 2008–February 2010	RCT	Total: 47 TIP: 23 GTIP: 24	TIP: 3.2 years GTIP: 3.5 years	Glanular to proximal penile hypospadias	2–5 years (mean 3.2 years)	UCF, MS, GD, meatus appearance, operative time
2	Helmy *et a*l^8^	Egypt	November 2012–November 2013	RCT	Total: 60 TIP: 30 GTIP: 30	TIP: 39.1±15 months GTIP: 41.9±16 months	Distal (subcoronal and distal shaft)	1 year	UCF, MS, GD, operative time, urinary flow rate
3	Eldeeb *et al* 9	Egypt	March 2017–December 2018	RCT	Total: 60 TIP: 30 GTIP: 30	TIP : 12 (6–24 months) GTIP: 13 (5–24 months)*	Distal penile	24 months	UCF, MS, urethral stricture, wound or glans disruption, operative time
4	Sultan *et al* 16	Egypt	December 2018–June 2020	RCT	Total: 60 TIP: 30 GTIP: 30	TIP: 30 months (9–144 months) GTIP : 36 months (12–96 months)*	Primary hypospadias	6 months	UCF, MS, GD, urine flow rate, overall success
5	El Shazly *et al* 17	Egypt	June 2018–June 2019	RCT	Total: 50 TIP: 25 GTIP: 25	TIP: 23±14.8 GTIP: 37.8±48.9	Distal penile	6 months	Meatal location, meatal shape, urinary stream, erection, UCF, operative time, HOSE score
6	Changpei *et al* 20	China	June 2016–June 2018	RCT	Total: 78 TIP: 39 GTIP: 39	TIP: 22.92±15.17 GTIP: 20.18±13.51	Coronal, distal, mid-penile	TIP: 23.54±4.68 GTIP: 24.85±4.37	UCF, stricture, HOSE score, GD, urine flow rate, operative time
7	Ahmed *et al* 26	Egypt	January 2018–July 2019	RCT	Total: 110 TIP: 55 GTIP: 55	TIP:33.6±39.4 months GTIP: 23.1±14.6 months	Distal hypospadias	6 months	UCF, MS, HOSE score
8	Zeina *et al* 19	Egypt	January 2017–July 2020	RCT	Total: 60 TIP: 30 GTIP: 30	TIP: 12.6±3.64 months GTIP: 11.40±3.10 months (range: 6–18 months)	Distal hypospadias	3–6 months	UCF, MS, meatal position, operative time, meatal recession, cosmetic appearance, urine flow rate
9	Mohammed *et al* 18	Egypt	November 2015–December 2016	RCT	Total: 42 TIP: 20 GTIP: 21	Mean age 9 years (range: 2–16 years)	Distal and mid-penile hypospadias	6–10 months (mean 8 months)	UCF, MS, mean operative time
10	Patel *et al* 21	India	2020–2022	RCT	Total: 40 TIP: 20 GTIP: 20	Not mentioned	Glanular, coronal, subcoronal, distal penile without chordee	2 years	UCF, MS, diverticulum, wound infection, proximal stricture

We classified all procedures named under Snodgrass, classic Snodgrass and TIPU as TIP and classified the modification of the TIP procedure with grafts as GTIP, DIGU, Snodgraft, grafted Snodgrass and GTAS as GTIP for the purpose of making uniformity of the above-mentioned procedures.

*Median with range.

DIGU, dorsal inlay graft urethroplasty; F/U, follow-up; GD, glans dehiscence; GTIP, grafted TIP; MS, meatal stenosis; RCT, randomized controlled trial; TIP, tubularized incised plate; TIPU, tubularized incised plate urethroplasty; UCF, urethrocutaneous fistula.

**Table 2:** Study outcomes

SN	Study	Type of repair	Total (n)	UCF	MS	GD	Diverticulum	HOSE score	Wound infection	Operative time (min)	Qmax mL/s	Urethral stricture	Success rate
1	Mouravas and Sfoungaris^7^	TIP	23	2	6	1	0	–	–	92 (72–110)*	–	0	16/23
		GTIP	24	1	0	1	0	–	–	115 (100–136)*	–	0	22/24
2	Helmy *et al* 8	TIP	30	0	1	0	0	–	–	79±9	11.2±5	0	29/30
		GTIP	30	0	0	2	0	–	–	106±12	11.6±3	0	28/30
3	Eldeeb *et al* ^9^	TIP	30	1	1	0	0	–	–	78 (55–99)†	–	0	28/30
		GTIP	30	1	0	1	0	–	–	110 (80–140)†	–	0	28/30
4	Sultan *et al* 16	TIP	30	3	3	1	0	–	–	74±8.00	10.89±2.15	0	23/30
		GTIP	30	2	1	1	0	–	–	102.37±9.27	18.59±3.33	0	26/30
5	El Shazly *et al* 17	TIP	25	2	2	0	0	15.7	–	85.2±6.3	–	0	22/25
		GTIP	25	0	0	1	0	15.5	–	91.4±6.2	–	0	24/25
6	Changpei *et al* 20	TIP	39	2	3	1	0	13.43±1.09		73.81±7.62	6.81±1.41	0	33/39
		GTIP	39	2	2	0	0	14.70±1.10		95.91±10.80	9.91±1.50	0	35/39
7	Ahmed *et al* ^10^	TIP	53	3	2	0	0	15.6±0.55	0	85.2±6.3	–	0	48/53
		GTIP	54	0	0	2	0	15.4±1.09	0	91.4±6.5	–	0	49/54
8	Zeina *et al* 19	TIP	30	1	2	0	0	–	–	50.07±7.28	–	0	27/30
		GTIP	30	1	1	0	0	–	–	75.47±9.25	–	0	26/30
9	Mohammed *et al* ^18^	TIP	20	1	2	0	0	–	–	75	–	0	17/20
		GTIP	21	1	1	0	0	–	–	88	–	0	19/21
10	Patel *et al* 21	TIP	20	3	2	0	0	–	1	–	–	2	–
		GTIP	20	1	1	0	1	–	1	–	–	0	–

*Mean and range.

†Median with range.

GD, glans dehiscence; GTIP, grafted TIP; MS, meatal stenosis; TIP, tubularized incised plate; UCF, urethrocutaneous fistula.

### Risk of bias assessment

**Figure 2:**
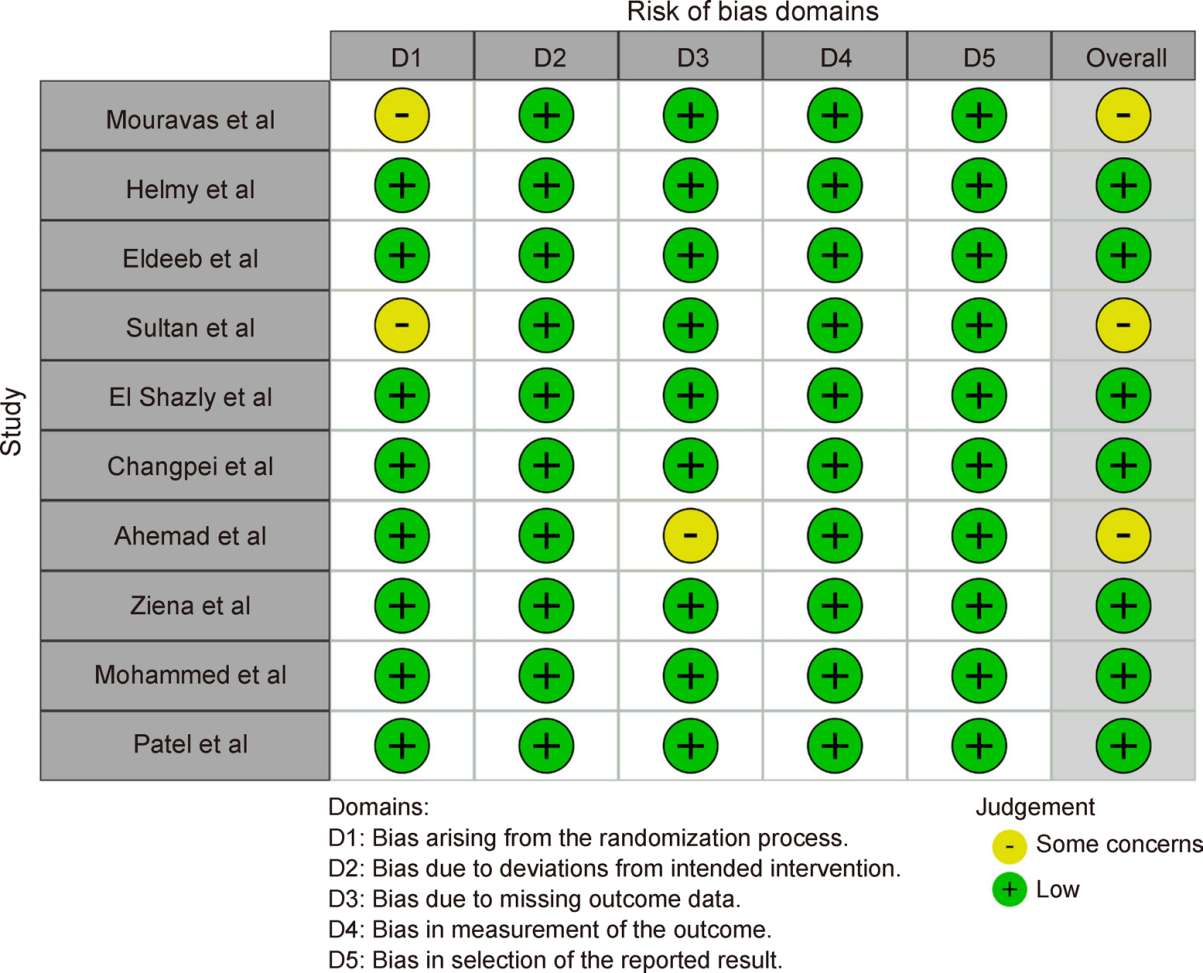
Risk of Bias Domains.

Risk of bias assessment (RoB 2) was performed across all the studies using the robvis tool and robvis web app (figure 2). Seven studies out of the 10 had an overall low risk of bias. However, for the remaining three studies, there were some concern in the domain of randomization^7 16^ and concern over missing outcome data.^10^

### Data synthesis and analysis of outcome

#### Urethrocutaneous fistula

**Figure 3:**
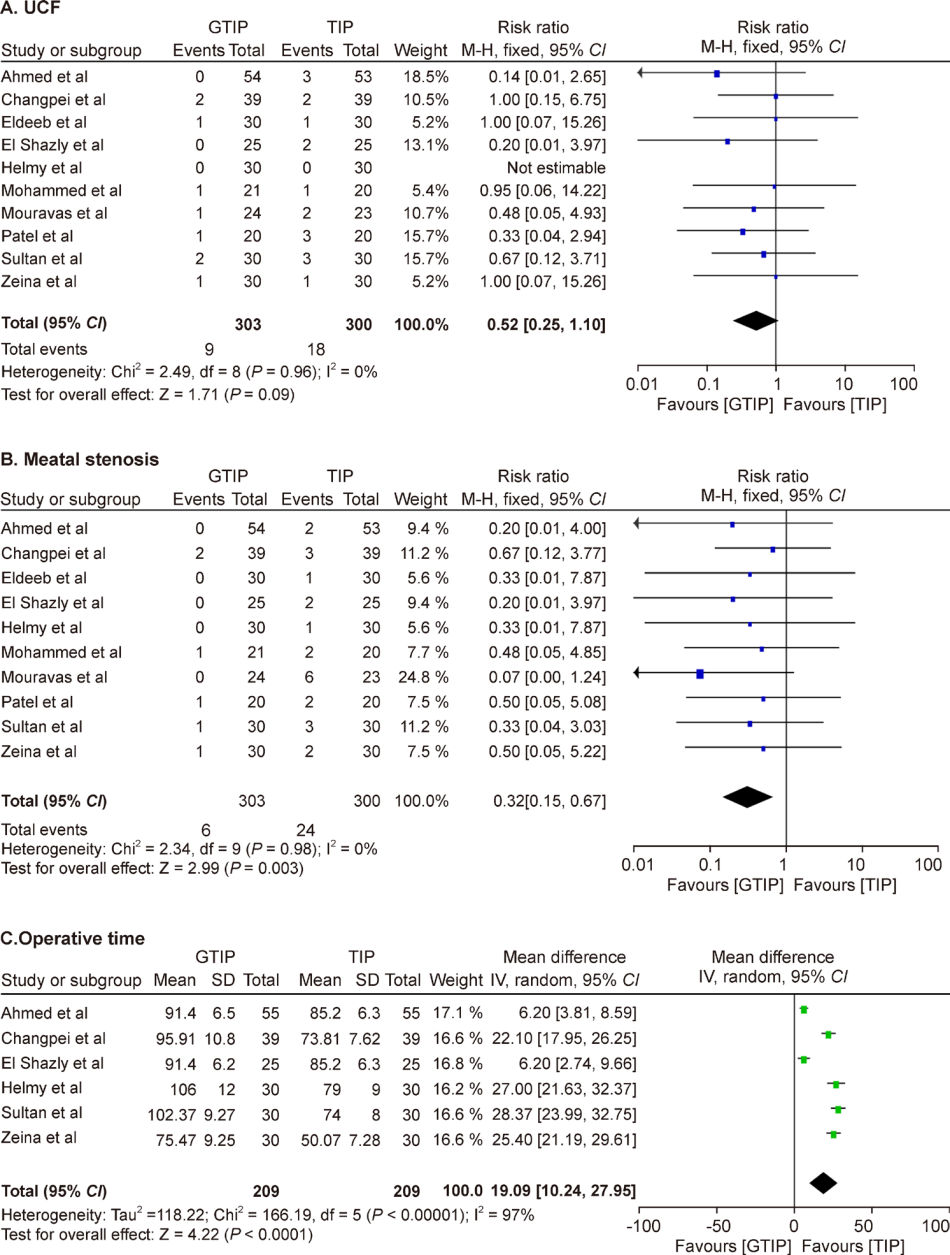
(A) Forest Plot (UCF) (B) Forest Plot (Meatal Stenosis) (C) Forest Plot (Operative Time). GTIP, grafted TIP; TIP, tubularized incised plate.

This outcome was reported by nine studies included in this meta-analysis. We calculated the incidence of UCF at the maximum follow-up period mentioned in the respective studies. There were nine UCFs in the GTIP group (2.97%) and 18 in the TIP group (6%). The study by Helmy *et al* 8 did not report any UCF events among the comparison groups. Pooled analysis of this outcome for the included studies showed that there was no statistically significant difference in the occurrence of UCF between the groups (RR=0.52, 95% CI 0.25 to 1.10), with no heterogeneity among the studies (I^2^=0%) (figure 3A).

#### Meatal stenosis

This outcome was reported by all 10 studies included in the meta-analysis. There were 6 events of MS in the GTIP group (2%) and 24 events in the TIP group (8%). Pooled analysis of the included studies for this outcome has shown a lower incidence of MS in the GTIP group (RR 0.32, 95% CI 0.15 to 0.67). No heterogeneity was observed for this pooled analysis (I^2^=0%) (figure 3B).

#### Glans dehiscence

This outcome was reported by seven studies included in the meta-analysis. Studies by Patel *et al*,^21^ Mohammed *et al*,^18^ and Zeina *et al* 19 did not report any event of GD in their reported series. There were eight events of GD in the GTIP group (2.64%) and three in the TIP group (1%). Pooled analysis of the included studies for this outcome showed that there was no significant difference in the occurrence of GD between the groups without heterogeneity (RR=1.89, 95% CI 0.68 to 5.24, I^2^=0%).

#### Operative time

All studies have reported this outcome except for Patel *et al*.^21^ Mouravas and Sfoungaris^7^ reported operative time as a mean with range, and Eldeeb *et al* 9 reported this outcome as a median with range. Mohammed *et al* 18 reported this outcome as a mean without range or SD. Ahmed *et al* 10 reported that a few of their patients failed to report for follow-up. For the meta-analysis of the outcome of operative time, we considered the total number of patients operated on by surgeons; however, for the analysis of the remaining outcome measures, we calculated the total number of patients who had completed follow-up. A pooled analysis of the remaining six studies was performed. Quantitative synthesis of this outcome showed that operative time was shorter in the TIP group than in the GTIP group, with significant heterogeneity (MD=19.09, 95% CI 10.24 to 27.95, I^2^=97%) (figure 3C).

#### Maximum flow rate

Only three studies reported this outcome.^8 16 20^ Pooled analysis of the three studies showed that the maximum flow rate was better in the TIP group than that in the GTIP group, with significant heterogeneity (MD=3.78, 95% CI 0.30 to 7.27, I^2^=95%). Although the maximum flow rate was in favor of TIP, as there were only a small number of participants in the three studies and high heterogeneity, the results need to be interpreted cautiously.

#### HOSE score

Only three studies reported this outcome. El Shazly *et al* 17 mentioned this outcome but did not provide the mean and SD values. Pooled analysis of the other two studies showed no significant difference in HOSE score among both groups, with significant heterogeneity (MD=0.52, 95% CI −0.92 to 1.96, I^2^=95%).

#### Success rate

Three of the included studies^9 10 16^ reported this outcome as a separate parameter. The study by Eldeeb *et al* 9 quoted success as having no complications. In the other two studies, the criteria for success were unclear. In the studies that did not provide the success rate as a separate outcome, we derived it after subtracting the complications from the total number of participants. In a study by Patel *et al*,^21^ we could not obtain the total number of complications, so the study was removed from the final analysis. Pooled analysis of the remaining nine studies for the outcome of success rate showed no significant difference between the groups, with heterogeneity (RR=1.02, 95% CI 0.97 to 1.08, I^2^=0%).

Other outcomes, such as diverticulum, wound infection and urethral stricture, have been reported by only one study each, so pooled analysis was not applicable. Nonetheless, these outcomes were not statistically significant in the described respective studies.

### Trial sequential analysis

**Figure 4:**
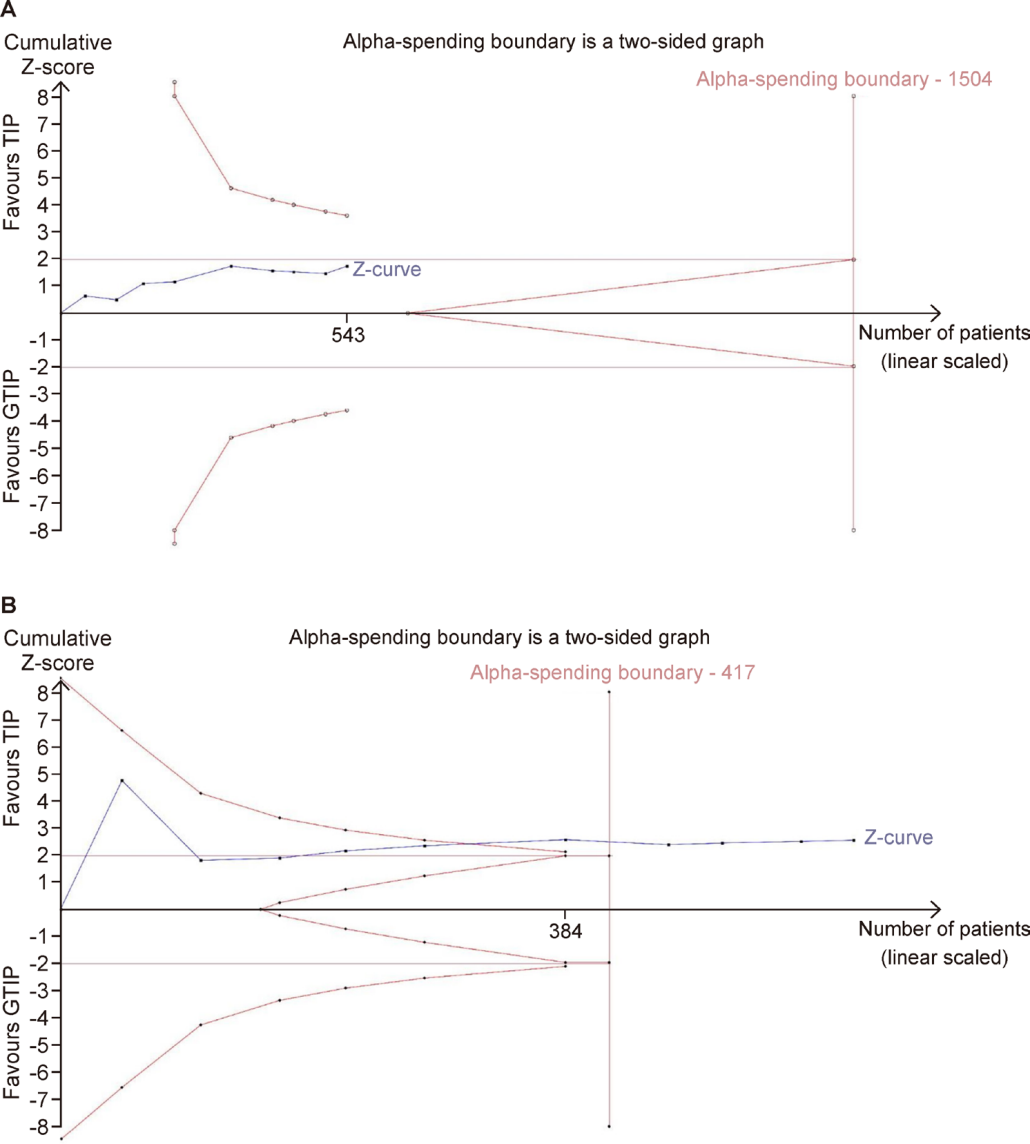
(A) Trial sequential analysis for UCF; (B) trial sequential analysis for MS. GTIP, grafted TIP; TIP, tubularized incised plate.

We performed TSA for the two primary outcomes, UCF and MS. The analysis of UCF shows that the information size of 603 was inadequate for the evidence to be conclusive, and it has not crossed conventional borders. The RIS for this outcome is 1504 (figure 4A). Analysis of MS showed that the cumulative Z-score crossed conventional boundaries and alpha-spending boundaries in favor of the TIP group. After reaching the RIS, the Z value remained greater than 1.96, making the meta-analysis of this outcome statistically significant (figure 4B).

## Discussion

Our literature search yielded 10 studies satisfying the eligibility criteria. There were 603 patients across the 10 studies used for our quantitative analysis. As per the quantitative analysis, supplemented by TSA, MS had a lower incidence in the GTIP group than in the TIP group. Our analysis also concludes that operative time is less in the TIP group than in the GTIP group. The other outcomes, such as UCF, GD, HOSE score, and success rate, were comparable in both groups.

The first case of TIP was performed by Warren Snodgrass in 1990 in a 9-month-old child who was planned for Mathieu repair.^22^ The rounded meatal appearance after Mathieu repair and the possible presence of hair follicles in the proximal margin of the outlined flap for Mathieu repair have incited the idea of TIP based on Rich *et al*’s^23^ principle of hinging of the urethral plate. In their own review of TIP, Warren Snodgrass and Nicol Bush mentioned UCF and GD are the most common complications encountered after TIP repair.^22^ They encountered only one case of MS among a series of 426 patients.^22^ A systematic review of complications rate of TIP in 3261 patients of distal hypospadias by Pfistermuller *et al* mentioned an overall incidence of 3.6% (1.7–7.4%) for MS, 5.7% (4.0–8.2%) for UCF and 1.3% (0.8–2.2%) for urethral stricture.^24^ The mean incidence of UCF (6.33%) in the TIP group in our pooled studies also falls within the range of the study by Pfistermuller *et al*,^24^ but the mean value of MS (8%) in our pooled data exceeds the range mentioned in that study. As mentioned earlier, Kolon and Gonzales^5^ modified the Snodgrass procedure by placing the graft at the bed of the midline incision of the TIP. In TIP, this midline incision of TIP heals through granulation and subsequent fibrous tissue, and the graft is covered with epithelium, minimizing fibrotic reaction, which is a possible explanation for many modifying TIP by placing the midline graft.^7 25^ The TIP procedure is frequently criticized for being unable to extend the midline incision to the tip of the glans beyond the urethral plate because of the possibility of scarring and consequent MS. According to proponents of the GTIP procedure, grafting distal incised glans and urethral plate enables the meatus to be appropriately positioned with a lower chance of MS. A case series of GTIP of 230 patients by Ahmed and Alsaid^26^ reported a UCF incidence of 3.91%, with no patients developing MS and urethral diverticulum in the postoperative period. A similar result was reported by Gupta *et al* 27 for 263 cases of GTIP with a 3.7% incidence of UCF with no reported cases of MS. Our meta-analysis findings regarding MS corroborate the two studies mentioned above; as there is a significantly reduced incidence of MS in the GTIP group supported also by TSA.

Our meta-analysis also yields a non-significant result for the outcome measure UCF between the TIP and GTIP groups. Similar results were also reported by retrospective evaluation by Shuzhu *et al* 28 and almost all other studies included in our quantitative analysis. This meta-analysis finding has been seconded by our TSA with the Z curve, although in the area of GTIP, it has not crossed conventional boundaries and has not reached the RIS. In our quantitative analysis, the outcome variable of operative time was less in the TIP group than in the GTIP group. The extra time taken for graft harvesting and fixing at the site of TIP usually accounts for an increase in the total duration of the procedure, which is the possible reason behind the prolonged operative time in the GTIP group. Although GTIP is a more time-consuming procedure, there was no difference in the cosmetic HOSE score between the groups. GD is also one of the complications that has been reported by a few authors included in our study and by Snodgrass and Bush.^22^ The difference between the groups for this outcome was not significant.

### Limitation

Inconsistent reporting of the outcome variables by the included studies is a limitation of this meta-analysis. For example, only a few studies have reported HOSE scores and urine flow rates as outcome variables. The result of hypospadias is usually dependent on the competency of the operating surgeon, which is challenging to access based on published reports. The variations in surgeon skill and experience could introduce additional sources of variability in the results and can sometimes make it challenging to draw certain conclusions. Assessment of MS can sometimes be subjective and is not uniform in the studies. This subjectivity may introduce uncertainty into the meta-analysis results and should be considered when interpreting the findings. The needed information size was also not reached for the outcome variable UCF on TSA.

### Strength

The strength of this meta-analysis is that all included studies are randomized controlled studies, and TSA has been used for the assessment of information size and statistical inference of a few primary outcomes.

### Conclusions

Our analysis has enough evidence to support a reduced incidence of MS in the GTIP group. Our meta-analysis also reveals that the needed operative time for TIP is less than that for GTIP. Both groups had no difference regarding other outcome measures, such as UCF, GD, HOSE score, and success rate. As TSA has substantiated our meta-analysis finding of MS only, we suggest further RCTs to attain the desired information size for the outcome of UCF. The outcomes in hypospadias repair may be influenced by the skill and expertise of the operating surgeon, which is often challenging to assess based solely on published reports. Additionally, the subjectivity in assessing MS across studies adds a layer of complexity to our findings.

## Data Availability

All data relevant to the study are included in the article or uploaded as supplemental information.
